# Medical News

**Published:** 1854-04

**Authors:** 


### THE MEDICAL EXAMINER.

PHILADELPHIA, APRIL, 1854.

### MEDICAL NEWS.

Dr. JOSEPH PANCOAST, Professor of Anatomy in the Jefferson College, has been elected Surgeon to the Pennsylvania Hospital, in the place of George Fox, M. D., resigned.

MEETING OF THE NEW YORK STATE MEDICAL SOCIETY.—The Society convened in its annual meeting, at the City Hall, in Albany, on the 7th of February, and continued in session till the afternoon of the 9th.

The Board of Censors appointed last year, at its previous meeting, to attend the examinations of the medical colleges in the city of New York, reported that the College of Physicians and Surgeons, in Crosby street, and the New York Medical College, in Fourteenth street, had invited and admitted them to their examinations of candidates for the degree of Doctor of Medicine. They reported that all the graduates of those schools were worthy of the degree.

From the University of the city of New York, in Thirteenth street, they reported that they had been refused the privilege or right of attending its examinations; that an answer from the faculty was deferred till a month after the time of graduation; and that, finally, a letter was received from professor Draper, assigning reasons for declining the supervision of the State Society. The letter was read and embodied in the report.

On the third day the matter was called up. A very general expression of the opinions of members was given. The course of the college was considered derogatory to the society, and subversive of all restraints in the conferring of degrees. A resolution was unanimously passed, censuring the college in strong terms, as being desirous of concealing its operations from the inspection of an honorable profession.

The Board of Censors were again appointed, with instructions to hold no intercourse with the University School unless specially invited so to do.—*Buffalo Med. Journal.*

A "Medical College of Virginia," governed by a board of nineteen visitors, appointed from different portions of the State, has been incorporated by the General Assembly of Virginia. The corporation is endowed with the property heretofore used for the purposes of the Medical School in Richmond, and with full powers over the faculty to be appointed hereafter.

---

The Boston Medical and Surgical Journal states that a Mrs. Frazer, of Slack Co., Ohio, has had six children within a single year, having had three at a birth twice.

---

The N. O. Medical and Surgical Journal, owned and edited by the late lamented Dr. Hester, has been sold since his death. It cannot fail, in the hands of its present able editor, Dr. Bennett Dowler, to give general satisfaction.

---

We have received the first number of the N. O. Medical News and Hospital Gazette, a semi-monthly Journal, edited by Drs. Samuel Choppin, C. Beard, R. Schlater and P. C. Boyer. We extract the following from their salutatory: "The connexion which our editorial corps has with the hospitals of New Orleans, affords the opportunity of presenting a regular report of interesting matter; and the assurances of assistance from other attending surgeons and physicians, warrant us in making the assertion that our pages will be found a mirror of hospital practice." We heartily wish them success in their laudable undertaking.

---

We have been much pleased with the perusal of "Doctors' Commons." The formation of libraries, pathological museums and cabinets of natural history, by State, County, and all local medical societies, is forcibly advocated by its author, Dr. S. W. Butler. If such a plan were generally adopted in every town and village, it would, independently of the kindly feelings engendered, tend greatly to the diffusion of knowledge and scientific inquiry among its members.

---

American Association for the Advancement of Science.—The next meeting of the Association opens at Washington on Wednesday, the 26th of April, and not on the 30th, as stated in the May number of the American Journal of Science.—*Eds. Am. Jour. Sci.*

---

Obituary.—Dr. George C. Shattuck, Sen., of Boston, aged 71

years. To the poor, the suffering and the struggling, Dr. S. was a benefactor and a friend. He was a man of great wealth, which, however, he seemed to prize only as he found occasion to use it nobly; of a heart full of kindness, and "a hand open as day for melting charity;" the best of neighbors, the best of citizens, the best of patriots; a steadfast lover and cherisher of all gentle and liberal and honorable things. It has never been our lot to know any one to whom could be more justly assigned the praise—

"Of that best portion of a good man's life,
His little, nameless, unremembered acts
Of kindness and of love."

*New York Churchman.*

At Columbus, on the 16th of January, Dr. Howard, in the 45th year of his age. Dr. H. was Professor of Surgery in Starling Medical College at the time of his death. As a teacher and operator he was deservedly celebrated.

We recorded in our February number the death of Dr. A. A. Gitenau, of Laurens County, Georgia. It should have been Dr. A. A. Giltenan.

